# On the Diversity of Malaria Parasites in African Apes and the Origin of *Plasmodium falciparum* from Bonobos

**DOI:** 10.1371/journal.ppat.1000765

**Published:** 2010-02-12

**Authors:** Sabrina Krief, Ananias A. Escalante, M. Andreina Pacheco, Lawrence Mugisha, Claudine André, Michel Halbwax, Anne Fischer, Jean-Michel Krief, John M. Kasenene, Mike Crandfield, Omar E. Cornejo, Jean-Marc Chavatte, Clara Lin, Franck Letourneur, Anne Charlotte Grüner, Thomas F. McCutchan, Laurent Rénia, Georges Snounou

**Affiliations:** 1 UMR 7206-USM 104, Eco-Anthropologie et Ethnobiologie, Muséum National d'Histoire Naturelle, Paris, France; 2 School of Life Sciences, Arizona State University, Tempe, Arizona, United States of America; 3 Chimpanzee Sanctuary & Wildlife Conservation Trust (CSWCT), Entebbe, Uganda; 4 Lola Ya Bonobo Bonobo Sanctuary, “Petites Chutes de la Lukaya”, Kimwenza–Mont Ngafula, Kinshasa, Democratic Republic of Congo; 5 Max-Planck Institute for Evolutionary Anthropology, Leipzig, Germany; 6 Projet pour la Conservation des Grands Singes, Paris, France; 7 Department of Botany, Makerere University, Kampala, Uganda; Makerere University Biological Field Station, Fort Portal, Uganda; 8 Research and Conservation Program, The Maryland Zoo in Baltimore, Baltimore, Maryland, United States of America; 9 Emory University, Program in Population Biology, Ecology, and Evolution, Atlanta, Georgia, United States of America; 10 USM0307, Parasitologie Comparée et Modèles Expérimentaux, Muséum National d'Histoire Naturelle, Paris, France; 11 Laboratory of Malaria Immunobiology, Singapore Immunology Network, Agency for Science Technology and Research (A*STAR), Biopolis, Singapore; 12 Institut Cochin, Université Paris Descartes, CNRS (UMR 8104), Paris, France; INSERM U567, Paris, France; 13 Laboratory of Malaria and Vector Research, National Institute of Allergy and Infectious Diseases, National Institute of Health, Bethesda, Maryland, United States of America; 14 INSERM UMR S 945, Paris, France; 15 Université Pierre & Marie Curie, Faculté de Médecine Pitié-Salpêtrière, Paris, France; 16 Department of Microbiology, National University of Singapore, Singapore; Washington University School of Medicine, United States of America

## Abstract

The origin of *Plasmodium falciparum*, the etiological agent of the most dangerous forms of human malaria, remains controversial. Although investigations of homologous parasites in African Apes are crucial to resolve this issue, studies have been restricted to a chimpanzee parasite related to *P. falciparum*, *P. reichenowi*, for which a single isolate was available until very recently. Using PCR amplification, we detected *Plasmodium* parasites in blood samples from 18 of 91 individuals of the genus *Pan*, including six chimpanzees (three *Pan troglodytes troglodytes*, three *Pan t. schweinfurthii*) and twelve bonobos (*Pan paniscus*). We obtained sequences of the parasites' mitochondrial genomes and/or from two nuclear genes from 14 samples. In addition to *P. reichenowi*, three other hitherto unknown lineages were found in the chimpanzees. One is related to *P. vivax* and two to *P. falciparum* that are likely to belong to distinct species. In the bonobos we found *P. falciparum* parasites whose mitochondrial genomes indicated that they were distinct from those present in humans, and another parasite lineage related to *P. malariae*. Phylogenetic analyses based on this diverse set of *Plasmodium* parasites in African Apes shed new light on the evolutionary history of *P. falciparum*. The data suggested that *P. falciparum* did not originate from *P. reichenowi* of chimpanzees (*Pan troglodytes*), but rather evolved in bonobos (*Pan paniscus*), from which it subsequently colonized humans by a host-switch. Finally, our data and that of others indicated that chimpanzees and bonobos maintain malaria parasites, to which humans are susceptible, a factor of some relevance to the renewed efforts to eradicate malaria.

## Author Summary

Chimpanzees and gorillas are known to have malaria parasites (genus *Plasmodium*) similar to those that infect humans. It is likely that detailed molecular studies of these parasites will help understand important aspects of the malaria disease and of immune defences in humans, and could then guide the development of novel control measures. However, few studies of parasites in African Apes have been conducted to date. Here we present the results of a survey of malaria parasites in chimpanzees and bonobos, our closest relatives. In chimpanzees, we identified two new parasite species closely related to *P. falciparum*, the most dangerous of the parasites in humans. We also found that bonobos harbour malaria parasites including *P. falciparum*. Phylogenetic analyses of these parasites strongly suggested that *P. falciparum* evolved in bonobos, and that it was introduced into humans from bonobos at a later date. Overall, our findings have substantially altered our perception of the origin of malaria parasites in humans.

## Introduction

Malaria infections have influenced the development of human civilizations, and have shaped the genetic make-up of current human populations. There are four globally distributed *Plasmodium* protozoan parasites that are responsible for malaria in humans (*P. falciparum*, *P. vivax*, *P. malariae* and *P. ovale*). Molecular phylogenetic analyses have demonstrated that these four parasites are not monophyletic [1],[2], indicating that they independently colonised hominids [3]–[6]. The timing of their appearance in *Homo sapiens*, however, remains unresolved. This is of some importance to current efforts to control malaria, because it will affect how observed patterns of genetic diversity in the parasite populations are interpreted. For example, several evolutionary genetic approaches rely on reliable phylogenetic information to detect putative adaptive genetic variation, thereby identifying genes that might be involved in pathogenesis or in the evasion of host immune responses. Addressing these issues is a matter of great importance for *P. falciparum*, the parasite responsible for a substantial proportion of the global malaria mortality and morbidity [7]. It is now generally accepted that *P. falciparum* underwent a population expansion in humans [4], [6], [8]–[11], though how, when and from where humans first acquired *P. falciparum*, is less well established. Suggestions of a host-switch from a chimpanzee parasite received recent support, albeit without resolving the likelihood or timing of this event [4],[10],[12].

The accuracy and robustness of conclusions derived from comparative analyses (phylogenetic or genomics) will be significantly enhanced if data from all of the evolutionary close parasites were to be included. In the context of parasites of humans, this data would be best obtained from *Plasmodium* species that infect our nearest relatives, the African Apes, because two of the parasite species, *P. reichenowi* and *P. rodhaini*, that have been reported in *Pan* and *Gorilla* are morphologically very similar to *P. falciparum* and *P. malariae* respectively, while the third, *P. schwetzi*, corresponds to *P. vivax* or *P. ovale* [13],[14]. Studies of the malaria parasites of African Apes have been limited to few observations made mainly in the 1920s–1950s, and very little is known of their natural history. Nonetheless, it is known that chimpanzees are susceptible to infection by the four parasite species of humans, while humans have been infected with *P. rodhaini* and *P. schwetzi* [13],[14]. The origin and evolutionary history of the malaria parasites in chimpanzees and gorillas are speculative [13],[14] mainly because the molecular data has been restricted to sequences derived from a single *P. reichenowi* isolate [3],[4],[8],[15],[16] until very recently [12]. In another recent publication, a novel parasite lineage close to, but distinct from, *P. reichenowi* was reported from chimpanzees sampled in Gabon [17]. This raises the important question as to whether *Plasmodium* species close to *P. falciparum*, other than the two described so far, occur in non-human higher primates.

We were afforded a rare opportunity to analyze blood samples collected independently from chimpanzees and bonobos for the presence of *Plasmodium* parasites. Such a collection of fresh isolates would provide sequence data for improved phylogenetic analyses. Here we report on our findings of a genetically diverse set of *Plasmodium* parasites found in some of the samples we analyzed, and we discuss the insights they have provided into the origin of the *Plasmodium falciparum*.

## Results

Blood samples were obtained from 49 chimpanzees, *Pan troglodytes*, in Uganda and the Democratic Republic of the Congo (DRC), and from 42 bonobos, *Pan paniscus*, in the DRC. Blood smears were not made available, so the presence and level of *Plasmodium* parasites were assessed solely by a highly sensitive PCR assay, where a small fragment of the small subunit ribosomal RNA (ssrRNA) genes is amplified using oligonucleotides that target sequences conserved in all known *Plasmodium* species [18]. Parasites were detected in 18 animals: 3/3 *Pan t. schweinfurthii* living wild in Kibale National Park in Uganda, and in 3/8 *Pan t. troglodytes* and 12/42 *Pan paniscus* cared for in sanctuaries in the DRC. Parasitaemias were quite low (<100 parasites per µl of blood), consistent with previous observations of naturally infected apes [13],[14].

We opted to conduct our analyses on the DNA purified directly from the blood samples, because whole genome amplification could lead to artefactual recombination between DNA molecules from different strains or species of parasites, should any be present in a given sample. Given the low parasite densities in the samples and the limited blood volumes available, efforts were directed at characterizing a small number of genes that have been used in recent phylogenetic analyses. Specifically, we targeted the mitochondrial genome using oligonucleotide primers that correspond to sequences conserved in *Plasmodium*. Since we were particularly interested in lineages related to *P. falciparum*, we used oligonucleotides based on sequences from *P. falciparum* to target two nuclear genes: dihydrofolate reductase-thymidylate synthase (*dhfr-ts*), and the gene encoding the merozoite surface protein 2 (*msp2*) because this gene is not known to have orthologues outside *P. falciparum* and *P. reichenowi* [15]. We specifically targeted the block 3 of *msp2*, because we hypothesized that the extensive polymorphisms observed for this region in *P. falciparum* might also occur in orthologous genes that could be present in closely related species, and this could provide an indication of genetic diversity in these parasites.

In order to minimize artefacts, nearly all the sequences obtained for the *dhfr-ts* and the *msp2* block 3 fragments were derived from duplicate amplifications. The mitochondrial genome sequences were also derived from duplicate amplification of a single 5800 bp fragment, which spans nearly the complete mitochondrial genome of ca. 6 kb. This avoided any ambiguities in a final assembly of overlapping fragments that might arise from a sample with multiple parasite lineages. Indeed, it was not possible to combine the *dhfr-ts*, *msp2* and mitochondrial data sets in the subsequent phylogenetic analyses, because mixed infections were common in our samples. Finally, we are confident that cross-contamination during amplification was highly unlikely because similar sequences for the different chimpanzee parasite lineages were derived from samples collected independently in Uganda or the DRC, and then processed in France or in the USA, respectively. Successful amplification was not achieved for all the genes targeted from each sample, and this was particularly noted for the samples from the bonobos. Nonetheless, the sequence data obtained revealed a rich diversity of species and strains (Table S1), in particular for the individual samples collected from the two *Pan troglodytes* subspecies.

**Figure 1:**
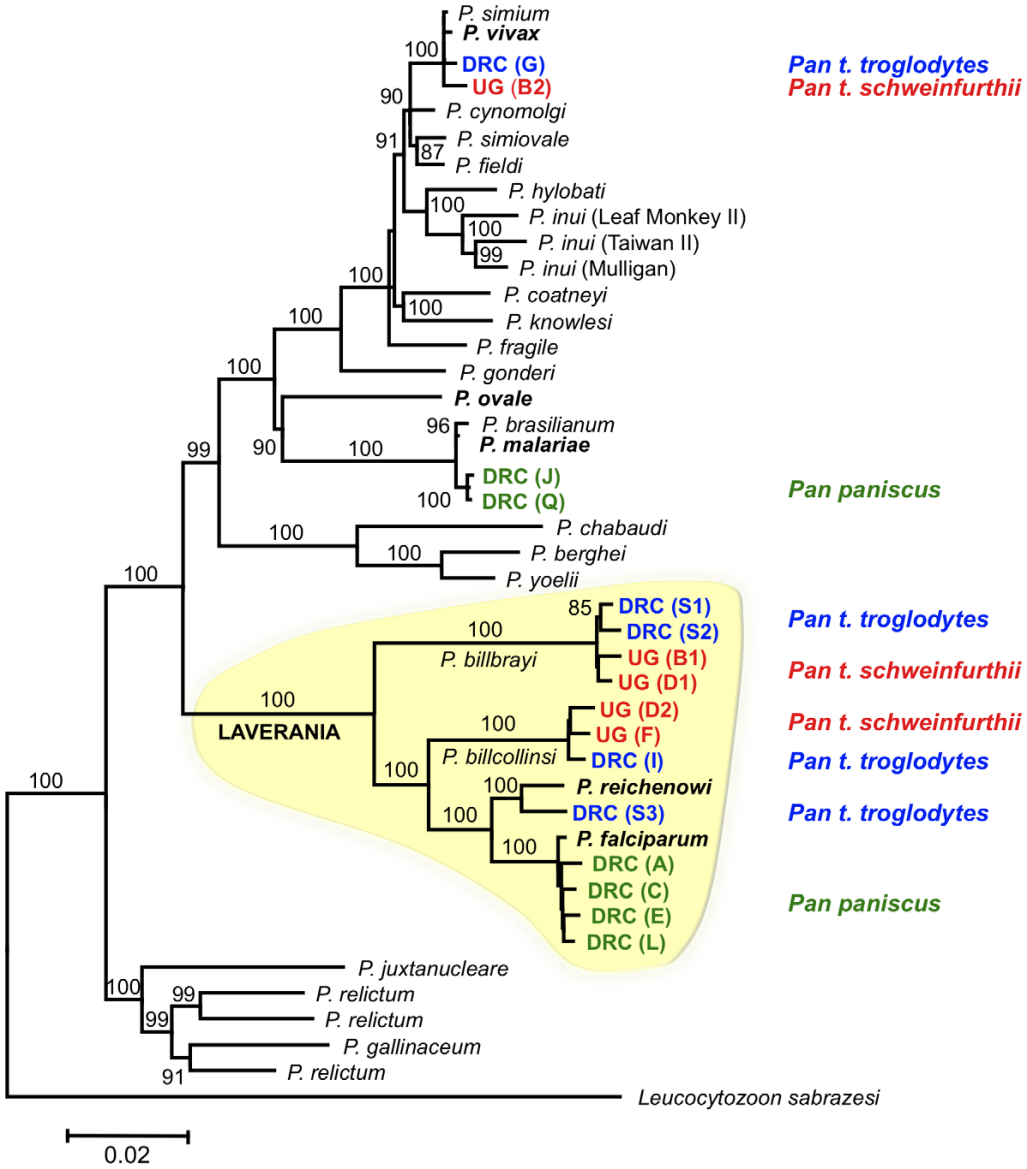
Phylogenetic tree of *Plasmodium* based on mitochondrial genomes. In the Bayesian phylogenetic tree presented, the values above branches are posterior probabilities expressed as percentages. Maximum likelihood and Bayesian methods lead to identical phylogenies. The names of the species that normally infect humans or chimpanzees are presented in bold. The sequence of the mitochondrial genomes derived from the Ape samples were named (also presented in bold) according to the country in which an Ape was sampled (DRC or UG, which stand for the Democratic Republic of Congo and Uganda, respectively), followed in parentheses by a single letter that indicates the particular Ape from which the sequence was obtained, and a number when two or more distinct sequence were obtained from the sample. Theses names were colour-coded according to the host species, indicated on the right, from which the sequences were derived (*Pan t. troglodytes* in blue; *Pan t. schweinfurthii* in red, and *Pan paniscus* in green). The Laverania clade is highlighted in yellow, and the branches carrying the sequences from the two novel lineages are labelled as the new species to which we propose they belong. The accession numbers of the sequences derived from the parasites found in chimpanzees and bonobos are provided in Table S1, and those of the other species are provided in the Methods.

**Figure 2:**
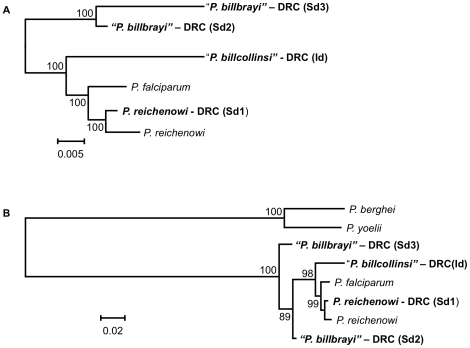
Phylogenetic analyses of the Laverania group based on the *dhfr-ts*. We report four *dhfr-ts* alleles, DRC (Sd1), DRC (Sd2) and DRC (Sd3) derived from the sample collected from one Ape (Shegue), and DRC (Id) derived from a sample from another Ape (Itaito). The DRC (Sd1) allele corresponds to the *P. reichenowi* sequence. In view of the similarity with the mitochondrial genome tree topology and the apparent lack of mixed species infection in the two animals from which sequences were obtained, we tentatively considered that DRC (Sd2) and DRC (Sd3) originate from *P. billbrayi* parasites, and DRC (Id) from *P. billcollinsi* (hence the quotation marks). Bayesian support for the nodes was inferred through a Monte Carlo Markov chain model as implemented in Mr. Bayes, with 10,000,000 generations after a “burn-in” of 3,000,000 generations. Sampling was performed every 100 generations. Mixing of the chains was properly checked after runs. Two phylogenies are presented for the gene encoding *dhfr-ts*. A. Phylogeny A (1789 bp), which included the *P. falciparum* (XM_001351443) and *P. reichenowi* (GQ369533, this study) *dhfr-ts* sequences and the four from parasites of Apes, reproduces the topology obtained from the mitochondrial genome. B. Phylogeny B (1690 bp aligned) uses rodent malarial parasites *P. berghei* and *P. yoelii* as outgroups, differs from the mitochondrial phylogeny by placing the root of the Laverania group within *P. billbrayi* alleles that are no longer monophyletic. We favour the phylogenetic hypothesis A over B since the latter is based on fewer base pairs and excludes an area with phylogenetic information among the Laverania species; such an area is not found in rodent or any other *Plasmodium* species so it is excluded from the phylogenetic analyses. Indeed, *P. reichenowi* (NC_002235 and DRC (Sd1)) cannot be clearly separated from *P. falciparum* indicating that rodent malarias may be too distant to serve as a reliable out-group for *dhfr-ts*.

**Figure 3:**
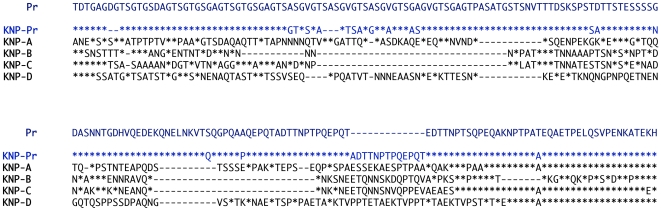
Alignment of the *msp2* block 3 sequences obtained from *Pan troglodytes* sp. The predicted amino acid sequence of one member from each of the five *msp2* block 3 allelic families uncovered from the *Plasmodium* parasites present in the chimpanzee samples. The alignment (Clusal V, DNASTAR Lasergene MegAlign version 7.2.1) comparisons were made against the only known *P. reichenowi msp2* block 3 sequence [15], denoted “Pr” (Y14731). The *msp2* block sequences obtained during our analysis were named according to the geographic origin of the samples “KNP” (Kibale National Park), followed by the sequence family (Pr for the *P. reichenowi* type in blue, and A to D for the others in black). Each distinct sequence found within each family was assigned a sequential number. The origins, names and accession numbers of all the *msp2* block 3 sequences obtained in this study are provided in Table S1. In the alignment presented the representative sequences from the five allelic families that were included are: KNP-Pr (Prmsp2-A1, GU075719), KNP-A (msp2-KNP-A1, GU075722), KNP-B (msp2-KNP-B, GU075724), KNP-C (msp2-KNP-C1, GU075725) and KNP-D (msp2-KNP-D, GU075726). Stars (*) represent residue similarity and dashes (−) represent gaps.

Sixteen near-complete mitochondrial genomes that coalesce in six distinct lineages were obtained from 12 of the 18 samples positive for *Plasmodium* (Fig. 1). All our phylogenetic analyses lead to identical topologies (see Methods), and only the Bayesian phylogenetic tree is reported (Fig. 1). Two lineages shared a recent common ancestor either with the *P. malariae* clade (two bonobos) or with the *P. vivax* clade (one chimpanzee from Uganda and one from the DRC). Another lineage, found in the bonobo samples, clustered with *P. falciparum*. One lineage from a DRC chimpanzee shared a recent common ancestor with *P. reichenowi*, while the two remaining lineages found in chimpanzees sampled in Uganda and the DRC, were novel and formed a monophyletic group with those of *P. falciparum* and *P. reichenowi*. For the sake of clarity, we have used the name Laverania to refer to this monophyletic clade, a generic name previously proposed to distinguish *P. falciparum* and *P. reichenowi* from the other malaria parasite species (International Commission on Zoological Nomenclature, Opinion 283). We hypothesized that the two new lineages in the Laverania clade correspond to two distinct *Plasmodium* species. This hypothesis was further supported by three other analyses. First, the extent of divergence in the genetic distances between these two novel Laverania lineages, as calculated from the mitochondrial genomes (Table 1), is comparable to that observed between well-established species in the rodent malaria clade, or between *P. falciparum* and *P. reichenowi*. Second, the topology of the phylogenetic tree constructed using *dhfr-ts* sequences from the same isolates reproduces that obtained for the mitochondrial genome (Fig. 2). Indeed, it would appear that an insert coding for eight amino acids is specific to the Laverania lineages (*P. falciparum*, *P. reichenowi* and the two new lineages), which further supports our conclusion that these lineages form a monophyletic group. Finally, the samples that harboured the two novel lineages and the *P. reichenowi* lineage, yielded *msp2* block 3 sequences that could be grouped into five distinct allelic families, of which one was similar to that previously published for *P. reichenowi* (Fig. 3), while the other four were novel. By a way of comparison, only two allelic families have been identified for the *P. falciparum msp2* block 3 despite extensive sampling.

**Table 1:** Genetic distances between the mitochondrial lineages (ca. 5800 bp) from selected *Plasmodium* species.

Species	*P. ber.*	*P. yoe.*	*P. cha.*	*P. fal.*	*P. rei.*	*P. bbr.* (S1)	*P. bbr.* (S2)	*P. rei.*(S1)	*P. bco.*
***P. ber.***	-								
***P. yoe.***	0.0132	-							
***P. cha.***	0.0308	0.0286	-						
***P. fal.***	0.0756	0.0747	0.0747	-					
***P. rei.***	0.0766	0.0761	0.0773	0.0104	-				
***P. bbr.*** ** (S1)**	0.0850	0.0832	0.0839	0.0434	0.0430	-			
***P. bbr.*** ** (S2)**	0.0860	0.0834	0.0846	0.0441	0.0437	0.0036	-		
***P. rei.*** **(S3)**	0.0796	0.0785	0.0785	0.0166	0.0115	0.0390	0.0397	-	
***P. bco.***	0.0836	0.0815	0.0820	0.0339	0.0348	0.0390	0.0397	0.0293	-

Species that infect rodents: *P. ber*  =  *P. berghei*; *P. yoe.*  =  *P. yoelii*; *P. cha*  =  *P. chabaudi*. Species that infect higher primates: *P. fal*  =  *P. falciparum*; *P. rei*  =  *P. reichenowi* (S3 in brackets indicated the haplotype DRC (S3) identified in this study). The new species described in this study are: *P. bbr.*  =  *P. billbrayi* (haplotype DRC (S1), DRC (S2)); *P. bcol*  =  *P. billcollinsi* (haplotype DRC (I)).

**Figure 4:**
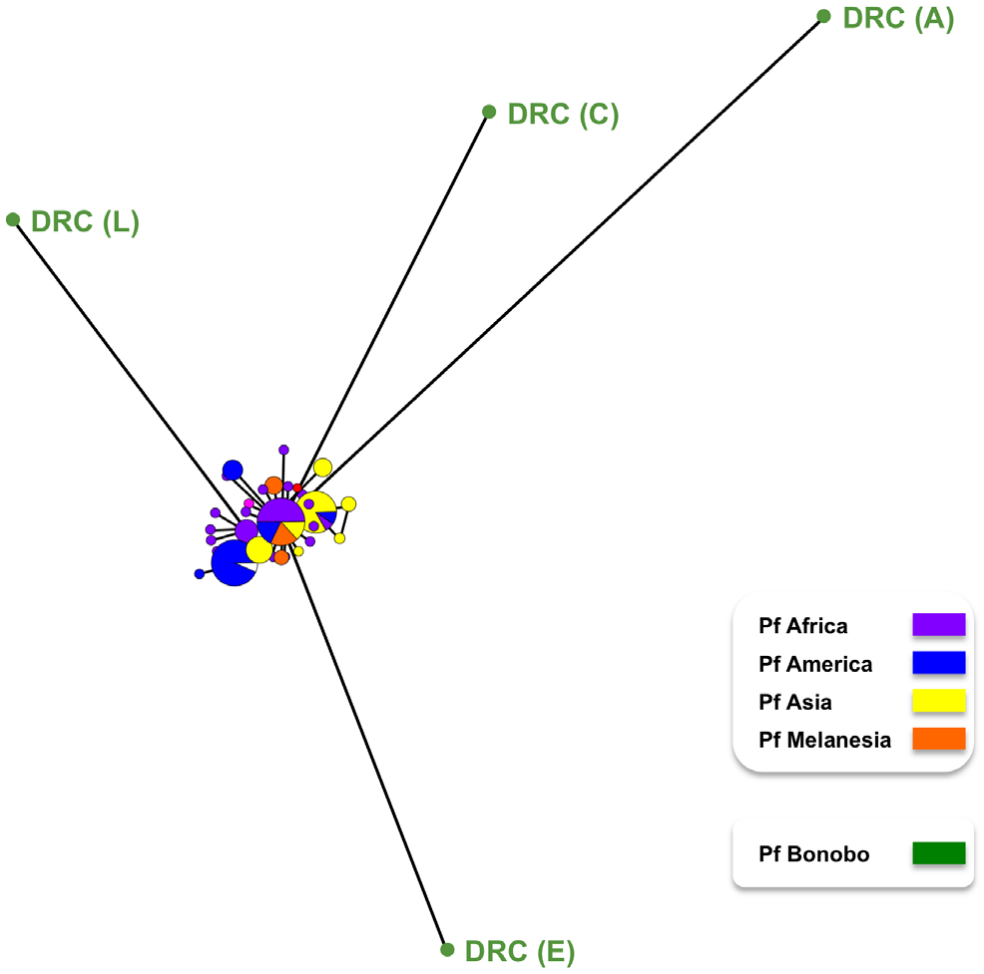
Mitochondrial haplotype map for *P. falciparum* populations found in humans and in bonobos. The mitochondrial genomes from parasite lines collected from humans [9] and of the four obtained from parasites in bonobos, DRC (A), DRC (C), DRC (E) and DRC (L), were used to obtain the haplotype network presented. It was inferred under a median joining algorithm with posterior pruning using maximum parsimony criteria as implemented in Network 4.1.1.2 [42]. The size of the circles is proportional to the haplotype frequency with each colour indicating which were derived from *P. falciparum* collected from bonobos, and the geographical origin of the sequences from *P. falciparum* collected from humans.

Six of the eight bonobos positive for *Plasmodium*, harboured parasites that yielded sequence data for *dhfr-ts* and/or *msp2*. The *msp2* and all the *dhfr-ts* sequences were indistinguishable from known *P. falciparum* sequences. This confirmed that bonobos were infected with *P. falciparum*, as had been indicated by the sequences of the mitochondrial genomes derived from four of these six bonobos (Fig. 1). Interestingly, we found significant differences in the genetic diversity of the *P. falciparum* mitochondrial lineages derived from bonobos as compared with that previously noted for large set of mitochondrial *P. falciparum* lineages obtained from human isolates collected worldwide [9]. Indeed, the *P. falciparum* lineages in bonobos (n = 4, π = 0.0048) were ten times more diverse that those found in humans (n = 96, π = 0.00034). Furthermore, there were no fixed differences between the *P. falciparum* from bonobos and those from humans. In other words, the four mitochondrial *P. falciparum* haplotypes we obtained from the bonobos had each a distinctive set of mutations such that none of these haplotypes were represented in the extensive *P. falciparum* mitochondrial haplotype database. This is clearly illustrated in the mitochondrial genome haplotype network (Fig. 4). The *P. falciparum* populations from bonobos and from humans, though related, have undergone some level of differentiation. Moreover, the haplotype network indicates that the four haplotypes from the bonobo do not form a monophyletic group, which suggests a scenario where bonobos and humans exchanged parasites in relatively recent times.

## Discussion

The sum of our knowledge on the *Plasmodium* parasites of African Apes derives from observations, nearly all made before the 1960s, on fewer than 50 naturally infected animals captured primarily in Cameroon, Sierra Leone or the Congo. Given the highly protected status of African Apes, prospects to extend this knowledge are restricted to molecular analyses of blood samples, mainly collected during medical examination of Apes cared for in sanctuaries, or upon recovery from poachers or villagers. The results from three such surveys published this year [12],[17],[19] have provided new glimpses into the diversity of malaria parasites in chimpanzees, and have allowed testing of hypotheses concerning the evolution of *P. falciparum* [12],[17],[19]. Here we present the outcome of two further independent surveys, one of which is distinguished by the inclusion of samples from bonobos and from wild-living chimpanzees. The molecular data we present demonstrate that the *Pan* genus naturally harbours a rich *Plasmodium* fauna, including two novel lineages close to *P. falciparum*, one related to *P. vivax*, and one related to *P. malariae*. Furthermore, it brings to light the presence of a population of *P. falciparum* in bonobos that appears to differ from those in humans. The observations add new perspectives to the evolutionary hypotheses formulated for the *Plasmodium* parasites of African Great Apes and humans.

From a parasitological point of view, the fact that the three samples collected from Eastern Chimpanzees (*Pan t. schweinfurthii*) living wild in a community of 44 animals, were all positive and harboured complex mixed strain/species infections (Table S1), suggests that prevalence of infections under natural conditions of transmission is high. This view is supported by our observations of a similar level of parasite diversity in three of the eight Central Chimpanzees (*Pan t. troglodytes*) that were independently sampled in the DRC (Table S1). It would be interesting to establish whether the other two chimpanzee subspecies, the Western Chimpanzee (*Pan t. verus*) and the Nigeria-Cameroon Chimpanzee (*Pan t. vellerosus*) also harbour the same parasite species. The bonobos cared for in a sanctuary also had high parasite prevalence, with *Plasmodium* detected via ssrRNA amplification in 12 of the 42 sampled (28.5%).

The parasites related to *P. vivax*-like found in chimpanzees from the DRC and Uganda might correspond to the chimpanzee parasite *P. schwetzi*. Preliminary evidence from partial *dhfr* sequences obtained for the chimpanzees we sampled in Uganda suggests that these parasites could be related to *P. vivax* (data not shown). Unfortunately, at present a *P. schwetzi* isolate is not available for comparative molecular analysis. Whether this species in *Pan* results from a past host switch from humans into chimpanzee, or whether it corresponds to *P. vivax* parasites recently reported in Equatorial Africa [20],[21], remains a matter of speculation. It might be that the dynamics of *P. vivax* and related species in African hominids, including humans, are more complex than previously thought.

The quartan malaria parasites, *P. brasilianum* in South American primates and *P. rodhaini* in the chimpanzee, have long been considered to be strains of *P. malariae* [13],[14]. Thus, it was interesting that the mitochondrial genomes of the parasites related to *P. malariae* found in two bonobos conform a sister clade and carry a six nucleotide insert that has not been observed for *P. malariae* or the South American parasite *P. brasilianum*. This could indicate that the parasites in bonobos might correspond to *P. rodhaini*, a species that would then be distinct rather than synonymous with *P. malariae*. Confirmation that this might indeed be the case awaits further molecular data from a larger set of *P. malariae* lineages from humans and Apes.

Three parasite lineages related to *P. falciparum* were found in both the chimpanzees collected from DRC and those collected from Uganda. One of these lineages clearly corresponds to *P. reichenowi*. We propose that the other lineages may represent two distinct *Plasmodium* species. Given the data from the near-complete mitochondrial genome sequences, and the support from *dhfr-ts* and *msp2* sequences, we consider it reasonable to ascribe specific status to the parasites in the two novel lineages observed in chimpanzees. We propose to name the parasites of one of the novel lineages *Plasmodium billcollinsi* Krief *et al.* n. sp., and those of the other *Plasmodium billbrayi* Krief *et al.* n. sp., in honour of the distinguished malariologists William E. Collins and “Bill” Robert Stow Bray (1923–2008), respectively. The type material would be the mitochondrial genome sequences (holotype and paratype), with a distribution in Uganda and the DRC in *Pan t. troglodytes* and *P. t. schweinfurthii* as hosts.

While we were finalizing this manuscript for submission, a publication describing a novel lineage related to *P. falciparum* was reported from two *Pan troglodytes* sampled in Gabon [17]. Based on mitochondrial DNA sequences, the authors have also proposed that this lineage be considered a new species, *P. gaboni* [17]. When the mitochondrial sequence submitted for *P. gaboni* was compared with the mitochondrial sequence presented here, it could be concluded that *P. gaboni* and *P. billbrayi* shared a recent common ancestor (Fig. S1). However, the differences were of sufficient importance (e.g. *P. gaboni* has a unique insert) to lead us to consider *P. gaboni* as a possible other additional member of the Laverania clade. Nonetheless, this assessment is at present mitigated by the fact that the contiguous mitochondrial sequence provided for the K isolate of *P. gaboni* (GenBank Accession No FJ895307) was assembled from discontinuous fragments that were amplified separately, hence the unavoidable gaps. Furthermore, if the animal from which the sample was obtained harboured a mixed infection, as did many of the chimpanzees that we sampled, the different fragments used for assembly might have originated from different species or lineages. Consequently, we opted not to consider the *P. gaboni* mitochondrial sequence in our phylogenetic analyses until such a time that the mitochondrial sequence from this lineage is confirmed, a view also adopted by Rich *et al.* [12].

We are aware that the validity of a species described only by sequences of one or more genes is open to debate, as this does not conform to current acceptable criteria. It would have been desirable to obtain some morphological data to provide a classical description of a novel species. The description of a new *Plasmodium* species is classically made after microscopic examination of Giemsa-stained infected erythrocytes, most often showing all asexual and sexual developmental stages. In some cases, it is necessary to examine the form of the parasite in the insect vector and/or during the hepatic stages, while for others differentiation from known species requires establishing one or more biological characteristics such as host specificity, the course of infection, or the ability to breed true. In the case of *Plasmodium* parasites that infect highly protected hosts (such as chimpanzees, gorillas and orang-utans) invasive sampling is highly restricted. On rare occasions it is possible to obtain a blood sample, but experimental infections of such animals are now nearly universally legally proscribed. Thus, the likelihood to obtain the morphological and biological data required to define and name a novel *Plasmodium* species for such hosts is highly remote. Furthermore, the presence in a single sample of multiple species would make it difficult to derive reliable conclusions from observations of a few blood smears. This is further exacerbated when parasite levels are low because this restricts microscopic examination to a few forms in thick smears where parasite morphology is poorly preserved. In our case, the six chimpanzees we sampled had low parasite loads, and four of them had mixed species infections. Had we had the opportunity to examine blood smears, a crescent-shaped gametocyte distinctive of *P. falciparum* and *P. reichenowi* might have been observed, but it would not have been possible to ascribe it with any degree of confidence to any one of the lineages detected by PCR amplification. Therefore, in the case of blood dwelling protozoan parasites of African Apes or other protected species, molecular data become the only accessible and reliable taxonomic features. In our study, we have considered that the phylogenetic analysis and genetic diversity comparisons based on the near-complete mitochondrial genomes, combined and supported with similar data from two nuclear genes, provided sufficient grounds to propose the description of two new species. The fact that similar sequence analyses correctly predict the specific status of well-established *Plasmodium* species (Fig. 1 and Table 1), adds to our confidence in the validity of *P. billbrayi* and *P. billcollinsi* as *bona fide* species. We nonetheless consider that it would be worthwhile for the community to agree on standardized parameters derived from defined molecular data that could serve to describe *Plasmodium* species for which no morphological or biological data are likely to become available.

The findings we present in this manuscript advocate a reappraisal of current views on the evolution and origin of *P. falciparum*. When it was thought that *P. reichenowi* and *P. falciparum* were unique among all primate malaria parasites, two hypotheses for the origin of *P. falciparum* as a parasite of humans were considered: co-speciation in their respective hosts, or a host switch followed by independent evolution. Grounds for favouring one hypothesis over the other shifted with time, as the weight of evidence that could support one hypothesis over the other was limited, principally by the availability of only a single *P. reichenowi* isolate. Recent analyses of data from parasites sampled from eight chimpanzees provided clear support for the host-switch scenario [12]. The data we present further support this finding and provide a more detailed account of the events leading to the origin of *P. falciparum* as a parasite of humans. When the tree topologies derived from the *dhfr-ts* and mitochondrial sequences (Fig. 1 & Fig. 2) are considered, the most parsimonious interpretation is that *P. falciparum* belongs to a monophyletic group of malarial parasites that have evolved in African Apes. We proceeded to estimate the divergence time of the most recent common ancestor for the Laverania clade. We agree that the use of molecular clocks is not without pitfalls, even when good time points can be used for calibration [22],[23]. In the particular case of parasitic organisms, an assumption of some level of host specificity (though not necessarily co-speciation) is needed in order to use host evolution for estimating the parasite mutation rates. Therefore, we estimated times of divergence of the mitochondrial sequences using models that allow the use of relaxed molecular clocks [24]. Although the *Homo*/*Pan* divergence time has been commonly used as a point of calibration for the *falciparum*-*reichenowi* divergence (e.g. [9],[17]), we excluded it in order to avoid circularity in the analyses. Thus, we estimated the mutation rates under two previously used scenarios: a), the *Plasmodium* spp. currently found in macaques radiated with their primary hosts, the genus *Macaca* [5], and b) *P. gonderi*, a parasite from African monkeys, and macaque parasites co-diverged when *Macaca* branched from other *Papionina* [25]. It is worth noting that neither of these two time points requires co-speciation (i.e. where specific malarial parasites co-speciate with specific non-human primate lineages generating phylogenies with identical topologies), but simply that several malarial parasites started their radiation with a major groups of non-human primates allowing for extensive host-switches. Such timeframes can be estimated even in the absence of good phylogenetic trees [26].

It is interesting that our time estimates (Table 2) that did not use the *Homo*-*Pan* divergence as a calibration point, were not substantially different from those estimated by others [9],[17] who used the *P. falciparum - P. reichenowi* divergence assuming co-speciation with *Homo*-*Pan*. The estimates of the divergence times for the Laverania clade members (Table 2) indicated that all the four lineages might have originated between 6.0 and 19 million years ago (Mya). Regardless of the wide confidence interval, this time frame is consistent with the origin of the genus *Pan*, but it clearly indicated that the Laverania lineages may have started to diverge long before the divergence *Pan*-*Homo* [27]. In addition, the phylogeny clearly indicates that the human parasite, *P. falciparum*, is the only *Homo* parasite among several *Pan* species in the Laverania clade. Given the phylogeny, a *Pan* host appears as an ancestral characteristic of the lineage. Therefore, when both phylogenies and estimated times of divergence are considered, a co-evolutionary origin of *P. falciparum* as a parasite of humans can be confidently excluded. Consequently the hypothesis that *P. falciparum* originated as a result of a host-switch between humans and Apes becomes favoured. However, our data indicate more complex scenarios that can only be addressed when data from multiple isolates of the parasite lineages currently present in both the hosts involved are included in the analyses.

**Table 2:** Estimated TMRCA for different parasite groups.

Clade	TMRCA mean Mya (L 95% CI–H 95% CI)
***P. falciparum*** ** in ** ***Pan paniscus***	0.77 (0.43–1.6)
***P. falciparum*** ** in ** ***H. sapiens***	0.20 (0.078–0.33)
***P. falciparum*** ** in ** ***Pan paniscus*** ** and ** ***H. sapiens***	0.85 (0.46–1.3)
***P. reichenowi***	1.8 (0.60–3.2)
***P. reichenowi*** ** - ** ***P. falciparum*** ** in ** ***Pan paniscus***	2.2 (1.0–3.1)
***P. billbray*** ** n. sp.**	1.1 (0.52–1.7)
***P. billcollinsi*** ** n. sp.**	0.97 (0.38–1.7)
**Laverania radiation**	12.0 (6.0–19.0)

The mitochondrial haplotype map (Fig. 4) provides evidence that the sub-population of four *P. falciparum* parasites in bonobos were genetically more diverse that of the extensive *P. falciparum* population in humans available to date. The most parsimonious interpretation of this line of evidence is that *P. falciparum* originated as a human parasite *via* a host-switch from *Pan paniscus*. When the human *P. falciparum* mitochondrial sequences alone are considered, our estimate of the time to the most recent common ancestor (TMRCA) was 78,000–330,000 years ago. While we cannot rule out that the available sample of *P. falciparum* mitochondrial genomes properly represent the genetic diversity of the species, this time frame is consistent with one expected for a parasite expanding early in human history. However, when considered together, the two distinct *P. falciparum* populations of humans and bonobos are estimated to have diverged from other members of the Laverania clade between 1.0 and 3.1 Mya. This timeframe coincides with the divergence of bonobo from the common chimpanzee [28],[29]. The estimated TMRCA of 0.4 to 1.6 Mya for the *P. falciparum* found in bonobos coincides with the origin of bonobos [28]. Taken together, our analyses indicate that *P. falciparum*, as a species, has long been associated with *Pan paniscus* and only subsequently switched into humans. The topology of the mitochondrial haplotype network (Fig. 4) is consistent with this interpretation and suggests that few lineages expanded in the human population after this event. The parasites we obtained over a short period from a single bonobo community probably constitute a biased sample set. A reliable estimate of the timing for the host-switch and the number of times this event might have taken place would require the inclusion of sequences from a larger set of *P. falciparum* parasites from bonobos from diverse locations. Assuming that there was no sampling bias with respect to the *P. falciparum* populations collected by others from humans, the limited data from bonobo parasites we present here can be most conservatively interpreted to support a single switching event, though it does not allow excluding multiple events. It is also possible that host switching still occurs today in areas where humans and bonobos are in close epidemiological contact. The presence of double or triple mutations associated with resistance to pyrimethamine in the four *dhfr* sequences obtained for the *P. falciparum* of bonobos is consistent with this, because these mutations are common in *P. falciparum* collected in 2008 from residents around Kinshasa [30]. At present, we cannot rule out the possibility that these *dhfr* mutations might have been selected independently in bonobos during the three months treatments with Bactrim™ (trimethoprim + sulfamethoxazole, two drugs that target the same enzymes of the folate pathway as the antimalarial combination of pyrimethamine and sulfadoxine) to which apes in the sanctuary were occasionally subjected. Finally, it could be speculated that the parasites in bonobos and in humans have recombined sexually.

The scenario we propose for the origin of *P. falciparum* in humans differs in several respects from a very recently formulated hypothesis that proposed that this species originated from a single transfer of *P. reichenowi* from chimpanzees to humans [12]. These conclusions were based on the analysis of the genetic diversity and tree topologies derived from fragments of the mitochondrial cytochrome b gene (528 bp), the apicoplast caseinolytic protease (316 bp), and the nuclear small subunit ribosomal RNA gene (371 bp), obtained from eight *Plasmodium*-infected chimpanzees (three from *Pan t. verus*, and five from *Pan t. troglodytes*). One assumption was that these sequences were derived from a single parasite specie, *P. reichenowi*, found in *Pan troglodytes* sp. This was a fair supposition to make since these short sequences did not provide sufficient resolution to distinguish their lineages from that of the only known *P. reichenowi* isolate. However, when these partial cytochrome *b* sequences are compared to the homologous region in the mitochondrial genomes that we obtained, there are clear indications that some might correspond to *P. reichenowi*, but also that most cluster either with the *P. billbrayi* or the *P. billcollinsi* lineages reported here (Fig. S1), which differ to such an extent from *P. reichenowi* that they could be considered as distinct species. Indeed this is evident on examination of the topology and branch lengths in the phylogenetic tree presented for the cytochrome *b* fragment (see Fig. 4 of [12]), where the eight isolates cluster into three groups removed from *P. reichenowi*. Our data provides evidence of a contrasting and more complex evolutionary scenario where *P. falciparum* evolved as a species in bonobos (*Pan paniscus*) where it was one of at least four parasite species that radiated in the genus *Pan* before it switched into humans.

The infections of bonobos by *P. falciparum* were not associated with any overt clinical signs, nor would the levels of parasitaemia have allowed detection by microscopy, suggesting a state of chronic malaria typical of infections in natural hosts. This is consistent with previous observations, including some made on splenectomised chimpanzees with high parasite levels [13],[14], in which chimpanzees experimentally infected with various parasite species including *P. falciparum* showed few clinical signs whether at peak parasitaemias or during the subsequent lengthy chronic infections [13],[14]. This minor impact on the health of chimpanzees was recently supported by the failure to detect a signature of positive selection in their G6PD genes, despite a long association with *Plasmodium* parasites [31]. The contrasting parasitological and clinical evolutions of *P. falciparum* in its two hosts, humans and bonobos, which have highly similar genomes, provides an excellent opportunity for comparative genomic studies to uncover the genetic or molecular basis for its higher virulence in humans. Such knowledge could be exploited to devise novel approaches to reduce the substantial global morbidity and mortality burdens.

It is likely that bonobos, in which we have found significant numbers to be naturally infected with *P. falciparum* or *P. malariae*, are also susceptible to infections by *P. ovale* and *P. vivax*, as is the case for chimpanzees [13],[14],[19]. One can now, therefore, justifiably explore whether bonobos and chimpanzees could act as a reservoir for all *Plasmodium* species that afflict humans. The potential impact of zoonotic malaria transmission on human health has been recently exemplified by a stable focus of potentially lethal *P. knowlesi*, a benign parasite of macaques, in inhabitants of Malaysian Borneo [32],[33]. Such a possibility has not been considered for sub-Saharan Africa. A zoonotic reintroduction of malaria into communities that live in hyperendemic areas is likely to be of little consequence. However, this would hinder efforts to eradicate malaria and might possibly lead to epidemic foci in formerly malarious regions whose inhabitants have lost immunity acquired against malaria. Furthermore, humans have been shown to be susceptible to infection by two of the parasite species of African Apes (*P. rodhaini* and *P. schwetzi*) [13],[14], and the meagre data available does not exclude the possibility that humans can be infected by *P. reichenowi* or the two new species we describe here. Using the sequence data we obtained from chimpanzee parasites, it will now be possible to seek these parasites in groups of humans that are in contact with African Apes.

In conclusion, the data gathered from a limited molecular analysis of a modest number of chimpanzee blood samples have not only significantly added to our knowledge of *Plasmodium* in our closest relatives, bonobos and chimpanzees, but also provided tantalizing insights into the evolutionary history of the malaria parasites of humans. We urge the scientific and the wildlife conservation communities to devote some resources to archive the parasites of Great Apes, which are at present likely to remain only amenable to molecular investigations, and to develop *in vitro* and/or *ex-vivo* methods to preserve and maintain them. These studies might provide novel approaches that could help control and eventually eradicate pathogens that have long exacted devastating global health, economic and social burdens.

## Methods

### Samples

#### Ethics statement

Collections of blood samples from animals in the DRC were made during routine annual medical check-ups. Authorization for the samples collected in the DRC was obtained from the Direction de la Conservation de la Nature et Organe de gestion de la CITES at the Ministère de l'Environnement, Conservation de la nature et Tourisme (DR), and the use of samples for scientific investigations was approved (CITES E0909/07). Specific authorization was also granted to “Les Amis des Bonobos du Congo” by the Ministère de la Recherche Scientifique (DRC). The few drops of blood from the chimpanzees at the Kibale National Park (Uganda) were obtained non-invasively: one in the course of post-mortem examination, the others from blood that dripped from wounds; collection of blood samples from the chimpanzees on Ngamba Island Chimpanzee Sanctuary (Uganda) were also conducted during routine annual medical check-ups; DNA extraction and preliminary PCR analysis were performed in Uganda. Authorization to use the DNA extracted from the samples for the purposes of genetic analyses of *Plasmodium* parasites that might be present was granted by the Uganda Wildlife Authority and the Uganda National Council for Science and Technology. The animal work was conducted according to relevant national and international guidelines. In all cases, the animals were not subjected to any experimental procedures, and the blood samples were obtained from aliquots collected independently by veterinarians carrying out routine medical examination. After consideration of the protocols of the study, the Arizona State University Institutional Review Board considered that the proposed molecular analyses of parasite DNA did not require formal approval. The Institutional Review Board of the Muséum National d'Histoire Naturelle also considered it unwarranted to seek formal approval for the genetic analysis of parasites present in material collected non-invasively and/or in an aliquot of samples collected during routine medical care of animals.

#### Chimpanzees, Uganda

Blood samples were collected on EDTA from three, wild, eastern chimpanzees (*Pan troglodytes schweinfurthii*), members of the Kanyawara community in Kibale National Park in western Uganda. A team led by S. Krief closely monitors the behaviour and health status of the Kanyawara chimpanzees. The blood samples have been opportunistically collected from one adult female (named NL) found dead on 20 Jan 2007, and from blood that dripped from wounds of an adolescent female (named JK) found caught in a snare on the 24 Oct 2006, and from those of another adolescent female (named OK) found injured on 30 Sep 2006. The samples were kept at −80°C until DNA extraction.

Blood samples were collected on EDTA in 2005 from thirty-eight semi-captive chimpanzees (*Pan troglodytes schweinfurthii*) at the Ngamba Island Chimpanzee Sanctuary situated on Lake Victoria close to Kampala in Uganda. The blood was collected under general anaesthesia during the routine annual health check monitoring. The samples were kept at −80°C at the Uganda Virus Research Institute (Entebbe, Uganda) until DNA extraction.

#### Chimpanzees, DRC

Eight orphan *Pan troglodytes troglodytes* from the DRC were sampled immediately after rescue between 2003 and 2006.

#### Bonobos, DRC

Blood samples were collected from 42 bonobos (*Pan paniscus*) that were kept at the Lola ya Bonobo Sanctuary, on the outskirts of Kinshasa in the Democratic Republic of Congo. The samples were obtained in 2007 as part of the routine annual health monitoring of 20 females and 22 males (age from 2 to 22 years old). The health status of each animal was scored on a scale of 1 to 3 (1 = good; 2 = medium; 3 = bad). 21 animals were scored 1, 18 were scored 2 and three were scored 3. Cough symptoms were noted in 19 individuals. Body temperatures ranged from 35.4°C to 37.7°C, but neither of these two parameters were correlated with the health score, nor with the presence of *Plasmodium* as detected by PCR. None of the animals suffered from diarrhoea, nor was blood found in the urine samples collected.

### DNA extraction, amplification protocols and sequencing strategies

For all samples, genomic DNA was extracted from aliquots of 200 µl of whole blood using the Qiagen DNeasy Blood and Tissue Kit (Qiagen, Germany), and the DNA obtained resuspended in 200 µl of buffer. Blood smears were not available for microscopic examination, thus parasite levels were estimated using PCR analysis of a 10-fold serial dilution series of the DNA purified from the positive samples. The nested PCR detection assay used was based on the small subunit ribosomal RNA gene (ssrRNA), using oligonucleotide primers that were specific to, and conserved in, all known *Plasmodium* species [18]. This established that the parasite burdens in these animals were very low (<10–100 parasite per µl of blood, and in one case <1000 parasites per µl).

Approximately 5,800 bp (out of 6,000) of the parasites' mitochondrial genome were amplified using the oligos Forward 5′-GAGGATTCTCTCCACACTTCAATTCGTACTTC and Reverse 5′-CAGGAAAATWATAGACCGAACCTTGGACTC with Takara LA Taq™ Polymerase (TaKaRa Takara Mirus Bio), (1 cycle 94°C for 1 min, then 30 cycles of 94°C for 30 sec and 68°C for 7 min− 1 cycle 72°C for 10 min). PCR products were cloned in the PGem®-T vector (Promega). In the case of the mitochondrial genome, we report sequences deposited in GenBank (Accession numbers are in parentheses following species name) for the Asian macaque parasites *P. inui* strain Taiwan II (GQ355483), *P. inui* strain Leaf Monkey II (GQ355482), and for *P. brasilianum* (GQ355484) from South American primates. Other sequences were reported in other studies: *P. inui* Mulligan (AB354572), *P. fieldi* (AB354574), *P. simiovale* (AB434920, AY800109), *P. knowlesi* (NC_007232), *P. cynomolgi* (AY800108), *P. fragile* (AY722799) and *P. coatneyi* (AB354575); *P. hylobati* (AB354573) from gibbons, *P. simium* (AY800110), *P. gonderi* from African monkeys (AY800111), and the parasites of humans *P. ovale* (AB354571) and *P. malariae* (AB354570). Additional information about these species, including their description, basic biology, geographic distribution and host-range can be found elsewhere [13]. Additional sequences of *Plasmodium* mitochondrial genomes were obtained from the GenBank (Accession numbers are in parentheses following species name): the avian malarial parasites *P. gallinaceum* (NC_008288), *P. juxtanucleare* (NC_008279), and *P. relictum* (AY733088–AY733090); the rodent malarial parasites *P. yoelii* (M29000), *P. berghei* (AF014115), *P. chabaudi* (AF014116); the non-human primate malarial parasite *P. reichenowi* (NC_002235); the human malarial parasite *P. falciparum* (AY282930) and *P. vivax* (AY598140). The avian parasite *Leucocytozoon sabrazesi* (NC_009336) was used as outgroup.

The gene encoding dihydrofolate reductase-thymidylate synthase (*dhfr-ts*) from *P. falciparum* or related species in samples collected from chimpanzees in Uganda and bonobos in the DRC was obtained as two overlapping fragments amplified by nested PCR using the following primer pair for the primary reaction: Pfdhfrts-F 5′-ATGATGGAACAAGTCTGCGACGTTTTCG and Pfdhfrts-R 5′-GCAGCCATATCCATTGAAATTTTTTCATG, (2.5 mM Mg^2+^, annealing at 58°C) The two separate secondary reactions were initiated with 1 µl of the product from the primary reaction using the following primer pairs Pfdhfrts-F and Pfdhfrts-NR 5′-GGGAAATATTGACTTAAATCAAATTTC (1.5 mM Mg^2+^, annealing at 58°C) that amplifies the fragment coding for the DHFR and linker domains, or Pfdhfrts-NF 5′-CAAAGTGATCGAACGGGAGTAGGTG and Pfdhfrts-R (3.5 mM Mg^2+^, annealing at 58°C) that amplifies the fragment encoding the TS domain. All reactions were initiated with 1 µl of template (equivalent to ca. 1 µl of whole blood) in a total reaction volume of 40 µl (final concentrations of 125 µM dNTP, 250 nM of each oligo, and 2 units/100 µl AmpliTaq polymerase), with the following cycling conditions: 95°C for 5 min, then 30 cycles of 2 min annealing (see above for temperatures used for each primer set), 2 min extension at 72°C and 1 min denaturation at 94°C, after a final annealing step followed by a 5 min extension step, the reaction temperature was brought down to 25°C before storage at −20°C.

The gene encoding the *dhfr-ts* from parasites related to *P. falciparum* in samples collected from chimpanzees in the DRC, was amplified using the primers: Forward 5′-ATGATGGAACAAGTCTGCG and Reverse 5′-TTAAGCAGCCATATCCATTG. The PCR conditions were: a partial denaturation at 94°C for 3 min and 35 cycles with 1 min at 94°C, 1 min at 53°C–55°C and 2 min extension at 72°C, a final extension of 10 min was added in the last cycle. Aligning *dhfr-ts* sequences among distantly related species of *Plasmodium* was difficult due to several insertions-deletions. We performed two analyses, one including only *P. falciparum*-like sequences on 1789 bp and a second including *P. gallinaceum* (AY033582), *P. chabaudi* (M30834), and *P. yoelii* (XM_719562) with only 1690 bp.

The fragment encoding the block 3 polymorphic domain of merozoite surface protein 2 (*msp2*) from *P. falciparum* or related species in samples collected from chimpanzees in the Uganda and bonobos in the DRC was by nested PCR amplification using the following primer pairs: primary reaction M2-P1 5′-GAAGGTAATTAAAACATTGTC and M2-P2 5′-GAGGGATGTTGCTGCTCCACAG, and a secondary reaction were initiated with 1 µl of the product from the primary reaction using M2-N1 5′-CTAGAACCATGCATATGTCC and M2-N2 5′-GAGTATAAGGAGAAGTATG. All reactions were initiated with 1 µl of template in a total reaction volume of 40 µl (final concentrations of 1.0 mM Mg^2+^, 25 µM dNTP, 250 nM of each oligo, and 2 units/100 µl AmpliTaq polymerase), with the following cycling conditions: 95°C for 5 min, then 30 cycles of 30 sec annealing at 50°C, 1 min extension at 72°C and 30 sec denaturation at 94°C, after a final annealing step followed by a 5 min extension step, the reaction temperature was brought down to 25°C before storage at −20°C.

In the majority of cases these sequences were derived from two or more independent amplifications. All the sequences obtained and reported here were submitted to GenBank (Accession numbers and the corresponding gene fragments are presented in the Table S1).

### Phylogenetic analyses

Initial Neighbor Joining (NJ) trees were inferred under Tamura-3P model of nucleotide substitution [34] in Mega4 [35]. Maximum likelihood (ML) search of a tree topology was implemented in PAML4 [36] under a General Time Reversible (GTR) + I + Γ_4_ substitution model, chosen based on likelihood ratio tests [37], and employing the NJ method to generate an initial tree. Bayesian support for the nodes was inferred in MRBAYES [38], under a General Time Reversible (GTR) + I + Γ_4_ substitution model, using 4 Markov chains and 10,000,000 Markov Chain Monte Carlo (MCMC) steps, discarding the first 3,000,000 steps (30%) as a burn-in. Sampling was performed every 500 generations. Mixing of the chains and convergence was properly checked after runs. The recovered ML and Bayesian trees were identical.

Although a total of eight distinct near-complete mitochondrial genomes were obtained from the parasites found in the bonobos, we stringently excluded any where the accuracy of the sequence obtained was not optimal, thus only 4 sequences were included in the phylogenetic and other analyses.

### Estimation of divergence times

The mutation rates that have been widely used in *Plasmodium* evolutionary genetic studies have used the *Homo*/*Pan* divergence time as a point of calibration for the *falciparum*-*reichenowi* divergence (for e.g. [9],[39]). However, using such rates will make whatever argument we put forward about the origin of *P. falciparum* and *P. reichenowi* circular. Thus, in order to avoid tautological arguments, we estimated mutation rates by considering time of divergence under two scenarios: i) the *Plasmodium* currently found in macaques radiated with the genus *Macaca* [5], which allows the estimation of a substitution rate of 2.83E-09 subs/site/year; ii) assuming that *P. gonderi* and macaque parasites co-diverged when *Macaca* branched from other Papionina [25], which allows the estimation of a mutation rate of 5.07E-09. It is worth noting that these mutation rates were not particularly off other estimates obtained for *Plasmodium* mitochondrial genomes (for e.g. [9]) indicating that, at least as first approximations, these scenarios are reasonable.

We employed a Bayesian approach with a relaxed clock [40] as implemented in BEAST [24]. The estimations of times of divergence for the clades of interest were performed by running 4 independent runs of 10,000,000 Markov Chain Monte Carlo (MCMC) steps after discarding the first 30% of the steps as burn-in, and sampling being performed every 1,000 steps. Previous runs showed that this burn-in was sufficient for the chains to reach stationary distribution. For the relaxed version of the clock we assumed a lognormal distributed clock for the mutation rate, with an average mutation rate according to each scenario mentioned in the previous paragraph, under a Yule prior for the simulation of the lineages during tree reconstruction. Results of the runs were analyzed with Tracer v1.4 [41] and estimates of average divergence times and confidence intervals were recovered. We checked the adequate mixing of the MCMC chains for each run in and the effective sample size of the estimates, making sure that all of them were above 100. The runs were combined in Tracer to generate the final estimates of time of divergence and their 95% confidence intervals.

### GenBank Accession numbers submitted with this manuscript

The following sequences were submitted to the GenBank: Near-complete *Plasmodium* mitochondrial genomes from parasites of chimpanzees, bonobos and other primate hosts GQ355468–GQ355486; *msp2* block 3 from parasites collected from *Pan t. schweinfurthii* (Uganda) GU075719–GU75726, and from parasites collected from *Pan t. troglodytes* (DRC) GU131994–GU131995; *dhfr-ts* sequences from parasites collected from *Pan t. troglodytes* (DRC) GQ369532–GQ369536; *P. falciparum msp2* block 3 sequences from bonobo samples GU075709–GU075718; *P. falciparum dhfr-ts* partial sequences from bonobo samples GQ859592–GQ859595).

## Supporting Information

Figure S1 Phylogenetic tree of *Plasmodium* based on a cytochrome *b* fragment. NJ tree on 520 bp of cytochrome *b* using Tamura 3 parameter model, 1000 bootstrap pseudo-replications. Haplotypes represented in bold are as follows: *Plasmodium* species that infect humans (black), the haplotypes we present in the manuscript (blue), the haplotype proposed as *P. gaboni* by Ollomo *et al.* [17] (purple), and the haplotypes presented as *P. reichenowi* by Rich *et al.* 2009 [12] (red). The species we propose, *P. billbrayi* and *P. billcollinsi*, are clearly set apart despite the relatively poor resolution inherent to using a short DNA sequence. For the “reichenowi-” haplotypes (red), “reichenowi-Rafiki1” and “reichenowi-Rafiki2” might belong to *P. reichenowi*, but three of the others cluster closely with *P. billcollinsi* and another three cluster more loosely with the *P. billbrayi/P. gaboni* group.
(0.35 MB PDF)

Table S1 Origin of the blood samples that yielded *Plasmodium* sequences (name in bold and GenBank Accession number in parentheses).
(0.09 MB PDF)
