# Supplementary material for: On the Diversity of Malaria Parasites in African Apes and the Origin of Plasmodium falciparum from Bonobos
Source: PLoS Pathog. 2010 Feb 12;6(2):e1000765. doi: 10.1371/journal.ppat.1000765 (PMC2820532; doi:10.1371/journal.ppat.1000765)
Supplement: Figure S1 — Phylogenetic tree of Plasmodium based on a cytochrome b fragment. NJ tree on 520 bp of cytochrome b using Tamura 3 parameter model, 1000 bootstrap pseudo-replications. Haplotypes represented in bold are as follows: Plasmodium species that infect humans (black), the haplotypes we present in the manuscript (blue), the haplotype proposed as P. gaboni by Ollomo et al. [17] (purple), and the haplotypes presented as P. reichenowi by Rich et al. 2009 [12] (red). The species we propose, P. billbrayi and P. billcollinsi, are clearly set apart despite the relatively poor resolution inherent to using a short DNA sequence. For the “reichenowi-” haplotypes (red), “reichenowi-Rafiki1” and “reichenowi-Rafiki2” might belong to P. reichenowi, but three of the others cluster closely with P. billcollinsi and another three cluster more loosely with the P. billbrayi/P. gaboni group. (0.35 MB PDF) [file ppat.1000765.s001.pdf]

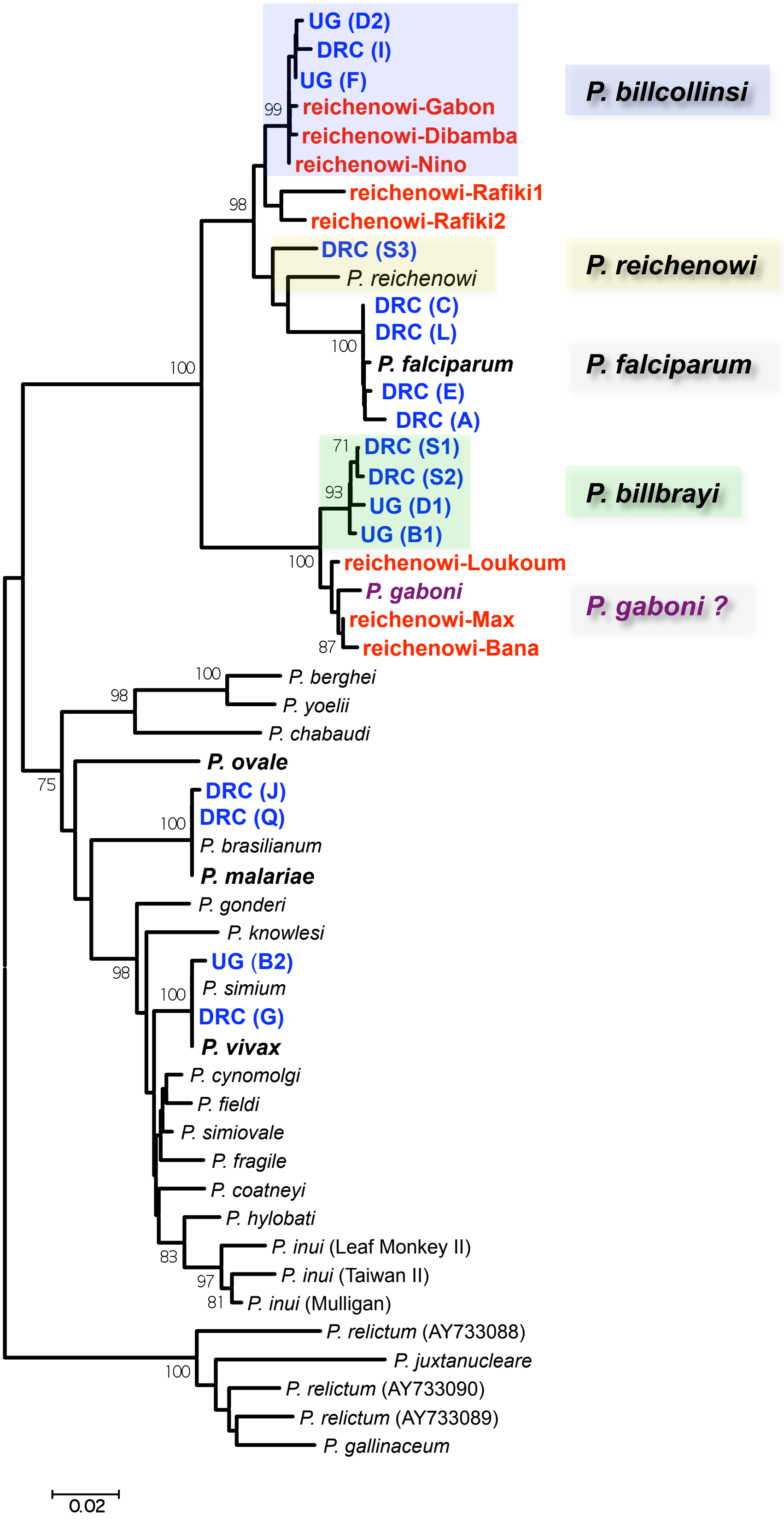

Fig. S1. Phylogenetic tree of *Plasmodium* based on a cytochrome *b* fragment. NJ tree on 520 bp of cytochrome *b* using Tamura 3 parameter model, 1000 bootstrap pseudo-replications. Haplotypes represented in bold are as follows: *Plasmodium* species that infect humans (black), the haplotypes we present in the manuscript (blue), the haplotype proposed as *P. gaboni* by Ollomo *et al.* [17] (purple), and the haplotypes presented as *P. reichenowi* by Rich *et al.* 2009 [12] (red). The species we propose, *P. billbrayi* and *P. billcollinsi*, are clearly set apart despite the relatively poor resolution inherent to using a short DNA sequence. For the "reichenowi-" haplotypes (red), "reichenowi-Rafiki1" and "reichenowi-Rafiki2" might belong to *P. reichenowi*, but three of the others cluster closely with *P. billcollinsi* and another three cluster more loosely with the *P. billbrayi* / *P. gaboni* group.
