# Supplementary material for: On the Diversity of Malaria Parasites in African Apes and the Origin of Plasmodium falciparum from Bonobos
Source: PLoS Pathog. 2010 Feb 12;6(2):e1000765. doi: 10.1371/journal.ppat.1000765 (PMC2820532; doi:10.1371/journal.ppat.1000765)
Supplement: Table S1 — Origin of the blood samples that yielded Plasmodium sequences (name in bold and GenBank Accession number in parentheses). (0.09 MB PDF) [file ppat.1000765.s002.pdf]

Table S1. Origin of the blood samples that yielded *Plasmodium* sequences (name in bold and GenBank Accession number in parentheses).

Country	Host Species	Ape Name	*P. falciparum*			*P. reichenowi*			*P. billcollinsi*			*P. billbrayi*		*P. malariae* type	*P. vivax* type
			mitochondrial	*Pfdhfr-ts* (partial 1041 bp) [a]	*Pfmsp2* block 3	*mitochondrial*	*Prdhfr-ts*	*Prmsp2* block 3	*mitochondrial*	*"Pbc"dhfr-ts* [b]	*Psp? msp2* block 3 [c]	*"Pbb"dhfr-ts* [b]	*mitochondrial*	*mitochondrial*	*mitochondrial*
**Uganda**	*Pan. t. schweinfurthii*	NL						**Prmsp2- A1** (GU075719)					**UG (B1)** (GQ355470)		**UG (B2)** (GQ355481)
**Uganda**	*Pan. t. schweinfurthii*	JK						**Prmsp2- A3** (GU075721)	**UG (D2)** (GQ355477)		**msp2-KNP-A1** (GU075722)<br>**msp2-KNP-A2** (GU075723)<br>**msp2-KNP-B** (GU075724)<br>**msp2-KNP-C1** (GU075725)<br>**msp2-KNP-D** (GU075726)		**UG (D1)** (GQ355471)		
**Uganda**	*Pan. t. schweinfurthii*	OK						**Prmsp2-A1** (GU075719)<br>**Prmsp2-A2** (GU075720)	**UG (F)** (GQ355478)		**msp2-KNP-A1** (GU075722)<br>**msp2-KNP-B** (GU075724)				
**Democratic Republic of the Congo**	*Pan t. troglodytes*	Gari													**DRC (L)** (GQ355480)
**Democratic Republic of the Congo**	*Pan t. troglodytes*	Itaito							**DRC (I)** (GQ355479)	**DRC (Id)** (GQ369534)					
**Democratic Republic of the Congo**	*Pan t. troglodytes*	Shegue				**DRC (S3)** (GQ355476)	**DRC (Sd1)** (GQ369532)				**msp2-KNP-A3** (GU131994)<br>**msp2-KNP-C2** (GU131995)	**DRC (Sd2)** (GQ369535)<br>**DRC (Sd3)** (GQ369536)	**DRC (S1)** (GQ355468)<br>**DRC (S2)** (GQ355469)		
**Democratic Republic of the Congo**	*Pan paniscus*	Dilolo			**FC27-C** (GU075712)										
**Democratic Republic of the Congo**	*Pan paniscus*	Kesa		**[N51I+S108N]** (GQ859592)											
**Democratic Republic of the Congo**	*Pan paniscus*	Kikwit	**DRC (L)** (GQ355475)	**[N51I+C59R+S108N]** (GQ859593)	**IC/3D7-C** (GU075718)										
**Democratic Republic of the Congo**	*Pan paniscus*	Lodja	**DRC (A)** (GQ355474)	**[N51I+S108N]** (GQ859594)	**FC27-A1** (GU075709)<br>**FC27-A2** (GU075710)<br>**IC/3D7-A1** (GU075714)<br>**IC/3D7-A2** (GU075715)<br>**IC/3D7-A2** (GU075716)										
**Democratic Republic of the Congo**	*Pan paniscus*	Opala	**DRC (C)** (GQ355473)		**FC27-B** (GU075711)										
**Democratic Republic of the Congo**	*Pan paniscus*	Mimia	**DRC (E)** (GQ355472)	**[N51I+C59R+S108N]** (GQ859595)	**FC27-D** (GU075713)<br>**IC/3D7-B** (GU075717)										
**Democratic Republic of the Congo**	*Pan paniscus*	Etumbe												**DRC (J)** (GQ355486)	
**Democratic Republic of the Congo**	*Pan paniscus*	Bill												**DRC (Q)** (GQ355485)	

[a] The non-synonymous mutations found occur in the codons of residues known to be associated with resistance to pyrimethamine *in P. falciparum*.

[b] The species to which the *dhfr-ts* sequence(s) was ascribed has been presumed. Confirmation that this is indeed the case awaits further samples with confirmed pure infections with either *P. billbrayi* or P. *billcollinsi*.

[c] At present it is not possible to ascribe any of these four *msp2* block 3 families to either *P. billbrayi* or *P. billcollinsi*.
